# Nectar Robbery by Native and Invasive Bumblebees Reduces Floral Rewards but Not Seed Production in *Desfontainia fulgens*

**DOI:** 10.1002/ece3.73933

**Published:** 2026-06-28

**Authors:** Carlos E. Valdivia, José I. Orellana

**Affiliations:** ^1^ Laboratorio de Vida Silvestre, Departamento de Ciencias Biológicas y Biodiversidad Universidad de Los Lagos Osorno Chile; ^2^ Instituto de Biología, Facultad de Ciencias Pontificia Universidad Católica de Valparaíso Valparaíso Chile; ^3^ Millennium Nucleus of Patagonian Limit of Life (LiLi) Valdivia Chile

**Keywords:** Chile, corolla perforation, floral larceny, foraging behavior, hummingbird pollination, south American temperate forests

## Abstract

Nectar robbery is common in hummingbird‐pollinated plants and is often assumed to reduce plant reproductive success by depleting floral rewards and disrupting pollination. However, its quantitative effects on plant fitness remain poorly resolved, particularly in systems where native and invasive nectar robbers coexist. We evaluated nectar robbery by the native bumblebee *Bombus dahlbomii* and the invasive *B. terrestris* in the hummingbird‐pollinated shrub *Desfontainia fulgens* in southern Chile. Flowering and nectar robbery were strongly synchronized, with robbery frequency peaking during maximum flower availability. Primary nectar robbing substantially reduced floral rewards: nectar standing crop in pierced flowers was approximately nine times lower than in intact flowers, whereas nectar sugar concentration showed only minor and statistically non‐significant changes. Small ants and flies were found inside the flowers, suggesting that these insects may act primarily as nectar thieves rather than effective pollinators. Their abundance was not significantly associated with corolla perforation. Floral visitation intensity differed among visitor species and foraging strategies, with bumblebees visiting more flowers per plant during nectar robbing than during legitimate pollination. However, these observations describe foraging modes rather than demonstrating that robbing itself caused a behavioral shift. The hummingbird *Sephanoides sephaniodes* was observed exclusively as a legitimate pollinator. Experimental manipulations showed no negative effects of nectar robbery on seed production when pollinators were present. Flowers exposed to pollinators produced substantially more seeds than flowers from which pollinators were excluded, confirming strong pollinator dependence. In contrast, both the probability of seed production and the number of seeds produced per flower were similar between flowers exposed to pollinators with and without nectar robbers. These results indicate that nectar robbery can markedly erode floral rewards without translating into reduced female reproductive output.

## Graphical abstract

We evaluated nectar robbery by native and invasive bumblebees in the hummingbird‐pollinated shrub *Desfontainia fulgens* in southern Chile. Nectar robbery strongly reduced nectar standing crop and altered floral visitation patterns, but these proximate effects did not translate into reduced seed production under natural pollination conditions. Our results show that floral larceny can markedly erode floral rewards without necessarily decreasing female reproductive output.

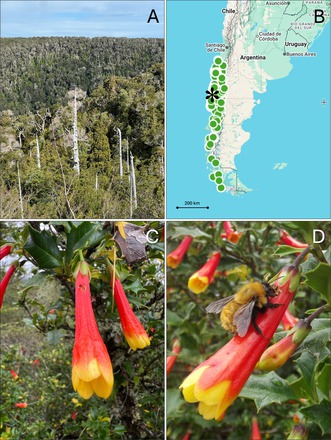

## 1 Introduction

Hummingbirds are highly efficient pollinators of numerous plant species across the Americas. In fact, more than 330 hummingbird species pollinate over 7000 plant taxa, from Alaska to Tierra del Fuego (Abrahamczyk and Kessler 2015). The selective pressures exerted by hummingbirds have strongly shaped floral morphology and rewards, leading many plants to exhibit tubular, elongated, red or yellow flowers with abundant nectar production (Wilson et al. 2006; Abrahamczyk and Renner 2015). These traits increase pollination efficiency but also restrict access by less effective nectarivores, such as short‐tongued insects (Wilson et al. 2006).

Some animals, mainly insects, can exploit nectar from hummingbird‐pollinated flowers without providing pollination services (Maloof and Inouye 2000; Irwin et al. 2010; Zhong et al. 2025). This behavior arises from morphological mismatches between flowers and visitors and promotes illegitimate foraging strategies. Nectar robbers perforate the corolla to access nectar (primary robbing) or extract nectar through holes made by other individuals (secondary robbing), whereas nectar thieves access nectar via the natural opening without damaging floral tissues or contacting reproductive organs (Inouye 1980).

The occurrence of nectar robbing is largely determined by the relationship between corolla length and visitor mouthpart length. Plants with longer and narrower corollas are more likely to be robbed, and visitors with shorter mouthparts more frequently adopt robbing behavior (Lara and Ornelas 2001; Urcelay et al. 2006; Valdivia et al. 2016). Nectar theft is favored when flowers have wide entrances and visitors are small enough to obtain nectar while avoiding contact with anthers or stigmas (Inouye 1980).

Floral larceny directly modifies floral rewards because nectar robbers and nectar thieves remove nectar without necessarily providing pollination services (Inouye 1980; Maloof and Inouye 2000; Irwin et al. 2010). These effects are commonly evaluated through the nectar standing crop, defined as the amount of nectar available in a flower at a given moment as a result of nectar secretion, evaporation, reabsorption, and removal by floral visitors (Corbet 2003; Pyke et al. 2020). In this sense, nectar standing crop represents the reward actually encountered by subsequent visitors rather than the total amount secreted by the flower (Corbet 2003; Pyke et al. 2020). Its main measurable attributes include nectar volume, sugar concentration, and, when chemically analyzed, sugar composition (Corbet 2003; Nicolson 2007; Pyke et al. 2020). Corolla perforation may reduce nectar volume directly by allowing robbers to extract nectar from the corolla tube, but it may also alter sugar concentration if perforations increase evaporation, change the timing of nectar depletion, or induce compensatory nectar secretion (Maloof and Inouye 2000; Irwin et al. 2010; Pacini and Nepi 2007). Therefore, changes in nectar standing crop provide a mechanistic link between illegitimate nectar removal and the behavior of legitimate pollinators (Irwin et al. 2010; Pyke et al. 2020; Leal et al. 2025).

Floral larceny has long been assumed to be antagonistic to plant reproduction, but its ultimate effects are context‐dependent and remain debated (Maloof and Inouye 2000; Irwin et al. 2010). Nectar robbery and nectar theft commonly reduce proximate floral traits, including nectar rewards, and can modify pollinator visitation (Maloof and Inouye 2000; Irwin et al. 2010; Leal et al. 2025; Zhong et al. 2025). However, reductions in nectar availability and changes in pollinator visitation do not necessarily translate into lower reproductive output, because the effects of floral larceny on fruit set and seed production range from negative to neutral or even positive (Maloof and Inouye 2000; Irwin et al. 2010; Andalo et al. 2019; Leal et al. 2025; Zhong et al. 2025). For example, Zhong et al. (2025) reported that floral larceny negatively affects proximate traits, pollinator visitation, and fruit set, while having weaker or neutral effects on other reproductive components such as seed set, seed quality, and male reproductive success. Similarly, Leal et al. (2025) showed that both nectar robbing and nectar theft reduce nectar rewards, although their consequences for pollinator visitation and plant reproduction differ between forms of larceny.

In the temperate forests of southern South America, hummingbird pollination is a distinctive and recurrent feature that originated approximately 16 million years ago and has evolved repeatedly across unrelated plant lineages (Smith‐Ramírez 1993; Abrahamczyk and Kessler 2015). In these ecosystems, the hummingbird *Sephanoides sephaniodes* functions as a key pollinator of numerous native plant species, including *Campsidium valdivianum*, *Fuchsia magellanica* , and *Embothrium coccineum* (Smith‐Ramírez 1993). These plants typically bear conspicuous, often pendulous, red to yellow flowers with elongated corollas and high nectar production, traits that enhance pollination efficiency but also increase vulnerability to illegitimate nectar foraging. Indeed, nectar robbing and nectar theft are widespread within these communities and are mediated primarily by native and invasive insects, and to a lesser extent by birds. Illegitimate nectar foraging has been reported in vines such as *Lapageria rosea* and *Campsidium valdivianum*; shrubs such as *Fuchsia magellanica* and *Mitraria coccinea* ; and trees such as *Embothrium coccineum* (Traveset et al. 1998; Gavini et al. 2022; Magrach et al. 2012; Rendoll et al. 2017; Urcelay et al. 2006; Valdivia and González‐Gómez 2006; Valdivia et al. 2016, 2018; Valdivia, Orellana, and Murúa 2025; Valdivia, Orellana, and Gantz 2025).

Preliminary observations in the Cordillera del Sarao, Chile, indicated that the shrub *Desfontainia fulgens* is mainly pollinated by *S. sephaniodes* but experiences primary nectar robbing by *Bombus dahlbomii* followed by secondary robbing by *B. terrestris* , an interaction not previously evaluated. We hypothesized that nectar‐robbing behavior by both bumblebee species would reduce nectar standing crop by removing nectar through corolla perforations and could alter sugar concentration if perforation promotes evaporation, changes the timing of nectar depletion, or induces compensatory nectar secretion. Because we did not analyze nectar sugar composition, our inference was restricted to nectar volume and total sugar concentration. We further hypothesized that nectar robbery would reduce seed production only if depleted rewards reduced effective pollinator service. The main aim of this study was to evaluate the effects of nectar robbing by *B. dahlbomii* and *B. terrestris* on a natural population of *D. fulgens* in southern Chile, while also characterizing the temporal dynamics of nectar robbery in relation to floral phenology. Specifically, we aimed to: (i) describe the flowering phenology of *D. fulgens* and the seasonal frequency of nectar robbing; (ii) determine robbing frequency and the effects of corolla perforation on nectar standing crop and sugar concentration; (iii) quantify visitation rates and foraging behaviors of the floral visitor assemblage; and (iv) assess the impact of nectar robbing on seed production.

## 2 Methods

### 2.1 Study Site and Species

**FIGURE 1:**
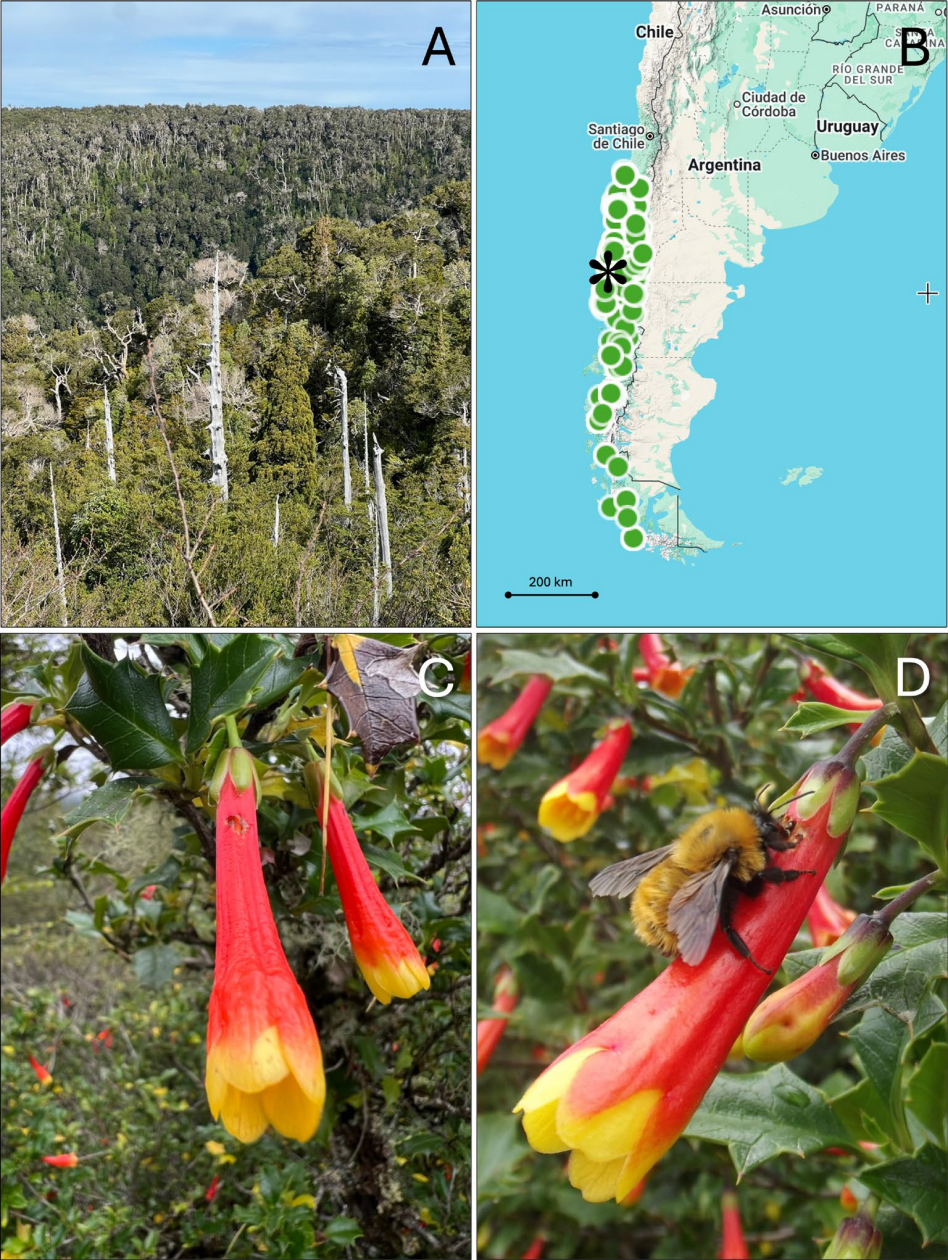
Study site, geographic context, and nectar robbery in *Desfontainia fulgens* . (A) Alerce ( *Fitzroya cupressoides* ) forest at the study site in southern Chile. (B) iNaturalist records used to estimate flowering phenology (green circles) and location of the study site (black asterisk). (C) *D. fulgens* flower showing characteristic nectar‐robbing damage. (D) The native bumblebee *Bombus dahlbomii* performing nectar robbery on a *D. fulgens* flower. Photo credits: Carlos E. Valdivia (A, C), José I. Orellana (D).

Fieldwork was conducted between February and November 2018 at the summit of the Cordillera del Sarao (867 m a.s.l.), Región de Los Lagos, Chile (40°56′S, 73°43′W) (Figure 1A,B). The climate at this site is classified as ultrahyperhumid and hyperoceanic, as moisture‐laden air masses from the Pacific Ocean ascend the coastal range and generate a pronounced rain shadow effect, resulting in high annual precipitation (1851–2162 mm) and low summit temperatures (6.7°C–7.9°C) (Luebert and Pliscoff 2023). The vegetation corresponds to the coastal temperate resinous forest dominated by *Fitzroya cupressoides* , a forest type that has been heavily exploited for timber and for non‐timber forest products such as *Sphagnum* moss (Luebert and Pliscoff 2023). The canopy is primarily composed of *F. cupressoides* (Cupressaceae), *Pilgerodendron uviferum* (Cupressaceae), *Nothofagus nitida*, and *N. betuloides* (Nothofagaceae). The understory includes numerous shrubs and climbing plants, several of which are hummingbird‐pollinated, including *Philesia magellanica* (Philesiaceae), *Berberis serrato‐dentata* (Berberidaceae), and *Desfontainia fulgens* (Collumeliaceae) (Luebert and Pliscoff 2023).

*Desfontainia fulgens* is a South American shrub distributed across temperate montane regions of Argentina (Provinces of Neuquén, Río Negro, Santa Cruz, and Tierra del Fuego) and Chile (Regions of Maule, Ñuble, Biobío, Araucanía, Los Ríos, Los Lagos, Aysén, and Magallanes), occurring from sea level up to approximately 1200 m a.s.l. (Rodríguez et al. 2018). In Chile, *Desfontainia fulgens* is a shrub reaching up to 2.5 m in height and bearing solitary, pedunculate flowers positioned in the leaf axils (Figure 1C; Muñoz 1980). The corollas are actinomorphic and infundibuliform, typically red but often displaying a yellow apical band on the petals (Figure 1C; Muñoz 1980). The species is self‐incompatible and therefore fully reliant on pollinators for fruit and seed production (Riveros et al. 1996). Its principal pollinator is the hummingbird *S. sephaniodes* , which visits *D. fulgens* flowers during the austral summer; however, nectar is also frequently robbed from these flowers by bumblebees (Fraga et al. 1997; Figure 1D). This species typically exhibits a late‐summer flowering peak, with anthesis concentrated between January and March, a pattern characteristic of several ornithophilous plants in temperate Andean forests. Such a phenological shift toward mid‐ to late summer reduces overlap with the flowering of non‐ornithophilous species and coincides with a period of sustained hummingbird activity, thereby enhancing opportunities for effective pollination (Aizen and Vázquez 2006). The fruit is a globose, greenish‐yellow berry approximately 1.4 cm in diameter, containing numerous small seeds of about 2 mm (Muñoz 1980).

### 2.2 Floral Phenology and Temporal Pattern of Robbing Events

On 1 December 2025, we downloaded all available observations of *D. fulgens* from iNaturalist (https://www.inaturalist.org), an online biodiversity platform where users upload georeferenced photographs and observations that can be curated for research use. Observations were filtered by plant taxon ( *D. fulgens* ) and country (Chile and Argentina), matching the filters reported in the data repository. We examined every photographic occurrence to assign phenological status, visually inspecting photographs to determine whether each plant was flowering. Out of 726 photographic occurrences of *D. fulgens* from Chile and Argentina, 537 records were retained as reliably flowering observations. This curation minimized errors associated with misidentifications, missing annotations, and observer inconsistencies. The retained flowering records were then examined for nectar‐robbing status. Robbing was scored only when the base or side of the corolla was sufficiently visible to determine whether a perforation was present. Photographs in which flowers were closed, only buds were visible, the flower base was obscured, or robbing status could not be determined with confidence were excluded from the robbery analysis and were not scored as unrobbed. This procedure yielded 537 flowering records with confidently assigned robbing status, of which 85 were classified as robbed.

To describe the flowering phenology of *D. fulgens* , we estimated the temporal distribution of flowering events using kernel density estimation (KDE), a non‐parametric method that provides a smooth representation of phenological curves and facilitates identification of the onset, peak, and decline of flowering (Silverman 1986; Wand and Jones 1995). To characterize the temporal pattern of robbing events, we calculated the monthly percentage of confidently scored flowering observations that showed signs of nectar robbery, allowing direct visual comparison between the seasonal distribution of flowering and the relative incidence of robbery. Because iNaturalist records are pooled across the geographic range of the species and do not provide complete monthly sampling for each population, these analyses were interpreted as a coarse‐scale description of seasonal synchrony rather than as a population‐specific estimate of robbery frequency.

To test whether the probability of nectar robbery changed linearly over the flowering season, we fitted a generalized linear model with binomial error distribution and logit link, using each confidently scored flowering record as the sampling unit, robbing status as the binary response variable (robbed vs. unrobbed), and month as the predictor. To evaluate whether nectar robbery tracked the seasonal availability of flowers, we assessed the association between the monthly number of flowering records and the monthly number of robbed flowers using Spearman's rank correlation. We did not fit an additional count regression to the same monthly data because this would duplicate the correlation analysis and because monthly counts are intended only as a descriptive, range‐wide measure of temporal association.

All statistical analyses were conducted in R version 4.3.2 (R Core Team, Vienna, Austria), and statistical significance was assessed at α = 0.05.

### 2.3 Primary Nectar Robbing and Floral Reward

To estimate the frequency of primary nectar robbing, we inspected ten mature flowers per plant on 100 plants (*n* = 1000 flowers). Flowers were visually examined for damage. Pierced flowers were recognized by the presence of a small hole or slit at the base or side of the corolla tube, consistent with perforations produced during nectar robbing (typically by *B. dahlbomii* ), whereas undamaged flowers showed no evidence of perforation. To minimize sampling bias, plant, branch, and flower selection were randomized: plants were selected within the population, branches were chosen at random within each plant, and mature flowers were selected haphazardly among available flowers without preference for size or condition.

To evaluate the effect of primary nectar robbing on floral rewards, we quantified nectar standing crop and sugar concentration in flowers exposed to natural visitation. Standing crop was defined as the amount of nectar available per flower at the time of sampling, reflecting the balance between nectar secretion and removal by nectar foragers (Corbet 2003). During peak flowering, we sampled 40 plants and selected ten mature flowers per plant (*n* = 400 flowers), comprising five undamaged and five pierced flowers per plant. Each flower was classified as pierced or undamaged, excised, and nectar was immediately extracted in the field using capillary tubes (75 × 1.5 mm). Nectar volume (μL) was estimated from nectar column length measured with a digital caliper (±0.01 mm). Sugar concentration was measured in the same nectar sample using a digital refractometer (Atago PAL‐1) and expressed as % w/w, whenever nectar volume was sufficient to allow reliable measurements (ca. ≥ 5 μL).

Because individual shrubs bore many open flowers, we did not census all flowers per plant. Instead, we calculated plant‐level mean nectar volume and mean sugar concentration separately for pierced and undamaged flowers (i.e., averaging the five flowers per category per plant), and used these paired plant means for inference to avoid pseudoreplication. When pierced flowers were present but completely depleted of nectar, nectar volume was recorded as 0 μL, as these values represent biologically meaningful nectar depletion.

Analyses of nectar volume were conducted using paired comparisons at the plant level (*n* = 40 plants), contrasting mean nectar volume between pierced and undamaged flowers within each plant. In contrast, analyses of sugar concentration were restricted to the subset of plants for which nectar volume was sufficient to measure sugar concentration in both pierced and undamaged flowers because nectar in pierced flowers was frequently absent or below the detection threshold. For each response variable, paired statistical tests were applied at the plant level. All analyses were conducted in R version 4.3.2 (R Core Team, Vienna, Austria), and statistical significance was assessed at *α* = 0.05.

### 2.4 Flower Visitors and Foraging Behavior

For the same flowers sampled for nectar standing crop and sugar concentration (five undamaged and five pierced flowers per plant; 40 plants), we assessed the presence of insects inside the corolla tube in the field at the Cordillera del Sarao study population during peak flowering in February–March 2018. Immediately after classifying each flower as undamaged or pierced and excising it for nectar extraction, the floral interior was inspected, and the number of insects present within each flower was recorded. Insects were identified to the finest taxonomic resolution possible using regional taxonomic keys, reference collections, and consultation with specialists; the taxa recorded were *Camponotus* sp. and *Lasiophanes* sp. (Formicidae) and *Dilophus* sp. (Bibionidae). The identity of the plant species was confirmed in the field based on vegetative and floral traits of *D. fulgens* .

To quantify rates of legitimate visits (pollination) and primary and secondary nectar robbing, we monitored the same 40 plants during peak flowering at the Cordillera del Sarao study population in February–March 2018. Each plant was observed during standardized 10‐min periods in the morning (08:00–12:00 h). Observers remained at approximately 3 m to avoid disturbing visitor behavior. Observations were conducted on sunny days to minimize weather‐related biases. During each observation period, we recorded the identity of floral visitors, the number of flowers visited per shrub, and the type of foraging behavior. Because observations began at 08:00 h, activity that may have occurred between sunrise and 08:00 h was not sampled; therefore, our visitation estimates apply to the standardized morning observation window rather than to the complete daily activity period.

Foraging behavior was classified following Inouye (1980), based on how visitors accessed nectar and their contact with reproductive structures. Visits were classified as pollination when visitors contacted the anthers and/or stigma. Primary nectar robbing was recorded when a visitor created a new perforation in an undamaged flower to access nectar, whereas secondary nectar robbing was recorded when nectar was extracted through an existing perforation. The number of visits per shrub, partitioned by foraging category (pollination, primary robbing, secondary robbing), was used to estimate visitation and robbing rates for each floral visitor taxon.

To evaluate the effect of floral perforations on the abundance of insects within the floral tubes of *D. fulgens* , and to test whether this effect differed among insect taxa, we analyzed insect counts per flower using generalized estimating equations (GEE), which extend generalized linear models to account for correlated observations. The response variable was the number of individuals per flower for each insect taxon. Floral status (pierced vs. undamaged) and insect taxon were included as fixed effects, along with their interaction. Because multiple flowers were sampled within each plant, plant identity was included as a clustering factor to account for non‐independence among flowers. Models were fitted assuming a negative binomial error distribution with a log‐link function, an exchangeable correlation structure, and robust standard errors. Population‐averaged estimates therefore refer to the expected number of insects per flower for each insect taxon and floral status across the sampled plant population. The significance of main effects and interactions was assessed using Wald chi‐square tests.

To evaluate differences in visitation intensity among floral visitor species and foraging strategies in *D. fulgens* , we analyzed the number of flowers visited per plant during standardized 10‐min observation periods using generalized linear models. Visitor species and foraging strategy (pollination vs. nectar robbing) were included as fixed effects, along with their interaction. Each plant represented an independent sampling unit. Due to overdispersion relative to a Poisson distribution, models were fitted assuming a negative binomial error distribution with a log‐link function. The significance of main effects and interactions was assessed using Wald *χ* ^2^ tests. Estimated marginal means and pairwise contrasts were calculated to facilitate biological interpretation of significant effects.

To evaluate whether nectar robbery or bee activity was associated with hummingbird visitation at the plant level, we conducted additional analyses using the same 40 focal shrubs monitored for floral visitors. We first tested whether the proportion of robbed flowers per plant predicted hummingbird visitation rate, using the number of *S. sephaniodes* visits per plant during the 10‐min observation period as the response variable in a negative binomial generalized linear model. We then tested whether total bee visitation predicted hummingbird visitation using the same model structure. Because hummingbird visits were rare, we also analyzed hummingbird visitation as a binary response variable, indicating whether each plant received at least one hummingbird visit during the observation period, using a binomial generalized linear model.

All analyses were conducted in R version 4.3.2 (R Core Team, Vienna, Austria).

### 2.5 Fruit and Seed Production

To evaluate whether primary nectar robbing by *B. dahlbomii* affected fruit and seed production in *D. fulgens* , while accounting for the role of pollinators, we established three experimental flower treatments on each of the same 40 shrubs used in the nectar measurements. Immediately before anthesis, we selected one flower bud per shrub for each experimental treatment and additionally marked one naturally open flower per shrub that already exhibited corolla perforations attributable to nectar robbing.

The first bud quantified fruit and seed production in the absence of floral visitors (−Pol − Rob). For this treatment, a single bud per shrub was bagged using fine tulle bags to prevent contact with pollinators and other floral visitors. The second bud quantified fruit and seed production in flowers accessible to pollinators but protected from nectar robbers (+Pol − Rob). To deter bumblebee robbing, masking tape was placed around the base of each bud, preventing bumblebees from biting the corolla. To minimize potential visual bias by legitimate pollinators, the masking tape was colored with indelible red ink to approximate the flower's natural coloration and allowed to dry completely before flowers opened. Although we did not detect visible avoidance or attraction to marked flowers, a potential olfactory effect of the ink cannot be entirely ruled out and is therefore acknowledged as a limitation. The third treatment quantified fruit and seed production under natural conditions, in flowers accessible to both pollinators and nectar robbers (+Pol + Rob). This treatment consisted of one naturally open flower per shrub that exhibited perforations consistent with primary nectar robbing by *B. dahlbomii* ; this flower was individually marked and left fully exposed to all floral visitors. The labels +Pol − Rob and + Pol + Rob indicate pollinator access, not confirmed pollination of each individual flower, because we did not directly observe pollination events on every experimental flower.

All experimental flowers were maintained under their respective visitor‐access conditions throughout the flowering and fruiting period (approximately 9 months). At the end of this period (November, austral spring), mature fruits were collected and processed to quantify fruit set and seed production. For each shrub, fruit and seed production were quantified at the flower level, with each shrub contributing one observation per treatment, allowing direct within‐shrub comparisons among the three pollination/robbing treatments. We did not quantify the total number of ovules per flower and therefore cannot report maximum seed set potential. Mature seeds were distinguished from undeveloped ovules or aborted seeds by their full size, firm structure, and dark seed coat; aborted seeds and undeveloped ovules were pale, flattened, or visibly shriveled and were not included in mature seed counts.

To evaluate treatment effects on seed production, we analyzed the number of mature seeds per flower using generalized linear models for count data. Because seed production exhibited a high frequency of zero values, reflecting flowers that failed to set seed, we applied a two‐part (hurdle) modeling approach that separated (i) the probability of seed production (seed presence vs. absence) from (ii) the number of seeds produced conditional on seed presence. The probability of seed production was analyzed using a binomial error distribution, whereas seed number conditional on production was analyzed assuming a negative binomial error distribution to account for overdispersion.

Pollination/robbing treatment (−Pol − Rob, +Pol − Rob, +Pol + Rob) was included as a fixed effect in both components of the model. Because treatments were applied within shrubs, plant identity was included as a clustering factor to account for non‐independence among observations from the same shrub. Model significance was assessed using Wald tests, and biologically relevant contrasts were used to explicitly evaluate the effect of nectar robbing under pollinator‐present conditions (+Pol + Rob vs. +Pol − Rob). All statistical analyses were conducted in R version 4.3.2 (R Core Team, Vienna, Austria).

## 3 Results

### 3.1 Flowering and Nectar Robbery Phenology

**FIGURE 2:**
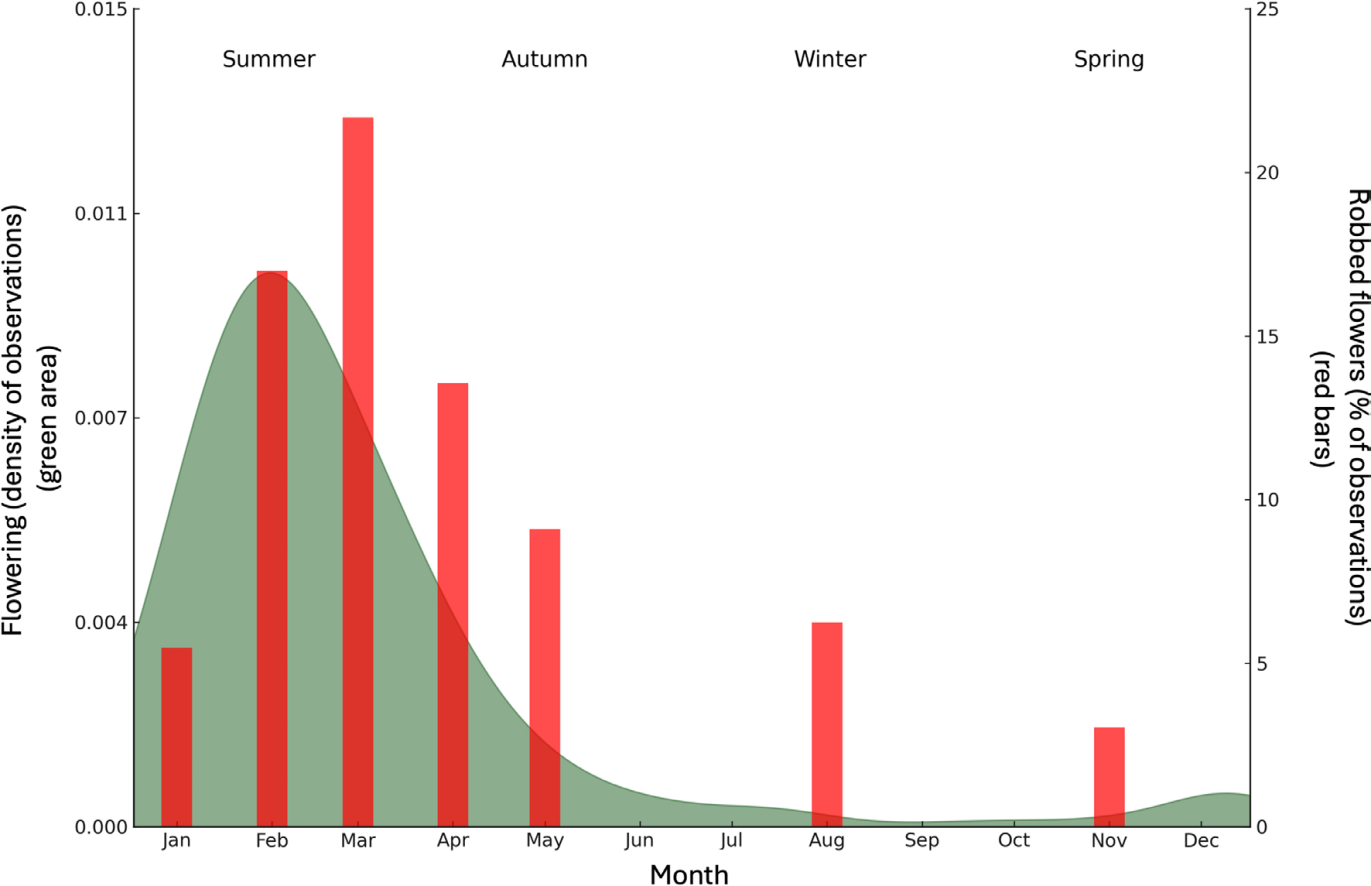
Flowering phenology and nectar robbery in *Desfontainia fulgens* . Monthly flowering density (green KDE curve) and proportion of nectar‐robbed flowers (red bars) based on curated iNaturalist observations. Flowering peaks in February–March, and nectar robbery shows a similar seasonal pattern, with highest incidence during the peak flowering period.

*Desfontainia fulgens* exhibited a pronounced summer‐autumn flowering pattern, with a sharp increase in flowering observations from January to March, a peak in February, and a progressive decline toward May. Only sparse records occurred outside this window (Figure 2). At the record level, the probability of nectar robbery did not show a significant linear change with calendar month (binomial GLM: coefficient for month = −0.047, *p* = 0.423). However, monthly flowering abundance was strongly correlated with the monthly number of robbed flowers (Spearman's rho = 0.766, *p* = 0.004), indicating temporal synchrony between flowering and nectar robbery at the range‐wide scale represented by the curated iNaturalist records. Because these records were pooled across populations in Chile and Argentina, this result should be interpreted as a broad seasonal pattern rather than as a population‐level estimate of flowering or robbery intensity.

### 3.2 Primary Nectar Robbing and Floral Reward

Across 100 plants (10 flowers per plant; *n* = 1000 flowers), 165 flowers showed corolla perforations consistent with primary nectar robbing, yielding an overall robbing frequency of 16.5% (Wilson 95% CI: 14.3%–18.9%). Robbing was heterogeneously distributed among plants: 43.0% of plants had at least one pierced flower (Wilson 95% CI: 33.7%–52.8%), and the plant‐level proportion of pierced flowers ranged from 0% to 100%.

**FIGURE 3:**
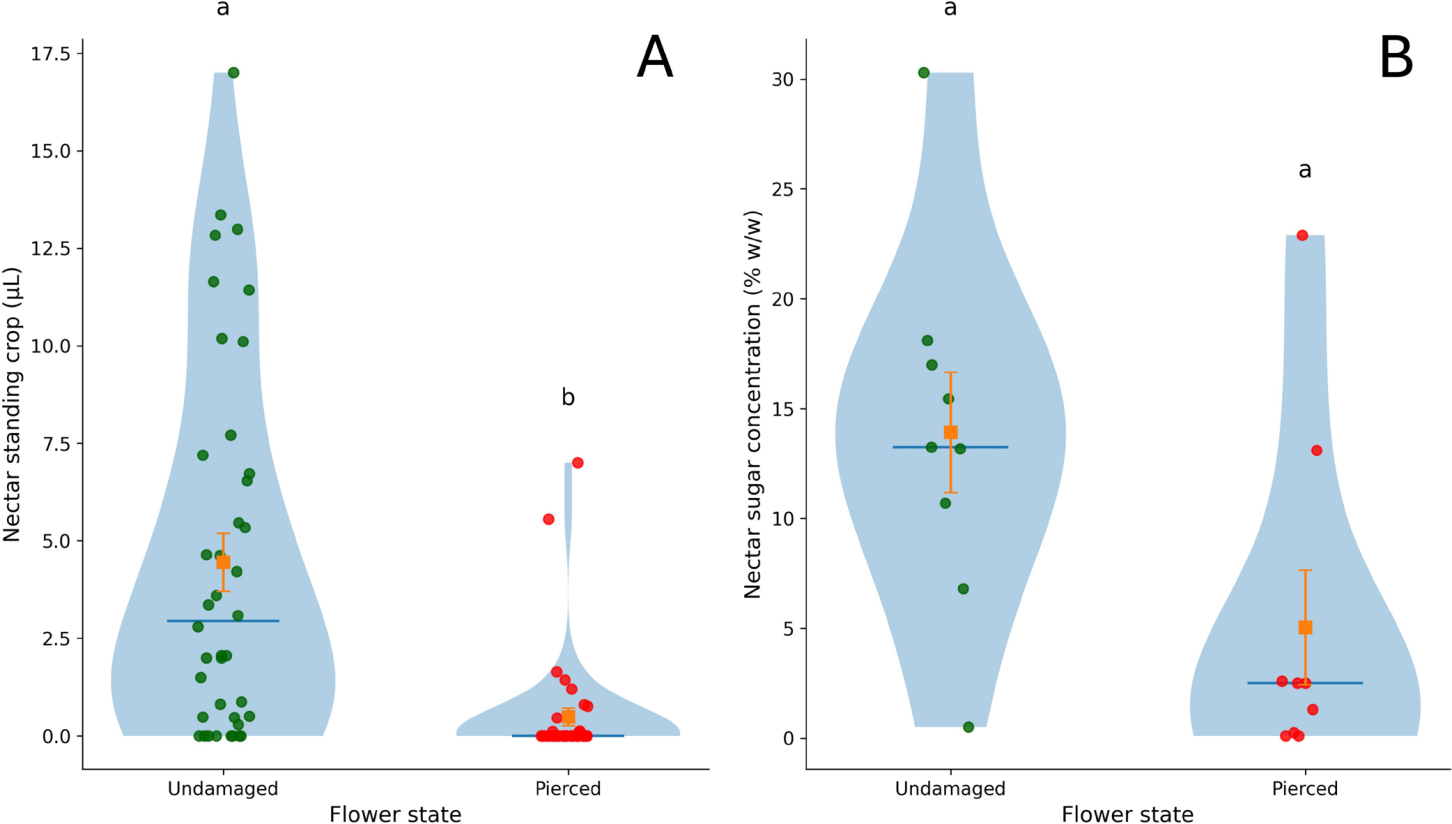
Effect of primary nectar robbing on nectar standing crop and sugar concentration in *Desfontainia fulgens* . (A) Violin plots showing the distribution of plant‐level mean nectar standing crop (μL) in undamaged and pierced flowers. (B) Violin plots showing the distribution of plant‐level mean nectar sugar concentration (% w/w), restricted to plants with measurable nectar in both flower states. Points represent plant‐level means, horizontal lines inside violins indicate medians, and squares with error bars show mean ± SE. Different lowercase letters indicate significant differences between flower states.

Using a paired plant‐level design based on mean values per shrub (averaging five pierced and five undamaged flowers per plant), nectar standing crop differed strongly between floral states. Across the 40 sampled plants, mean nectar volume in pierced flowers was approximately 9.25‐fold lower than in undamaged flowers, corresponding to an 89.2% reduction, and this difference was statistically significant (paired *t*‐test: *t*_39_ = 5.41, *p* = 3.39 × 10^−6^; Figure 3A).

In contrast, analyses of nectar sugar concentration were restricted to plants for which measurable nectar was available in both pierced and undamaged flowers. This resulted in a reduced sample size of 9 plants. Within this subset, mean sugar concentration in pierced flowers was approximately 2.76‐fold lower (63.8%) than in undamaged flowers. However, this difference was not statistically significant (paired *t*‐test: *t*_8_ = −2.13, *p* = 0.066; Figure 3B).

### 3.3 Flower Visitors and Foraging Behavior

**FIGURE 4:**
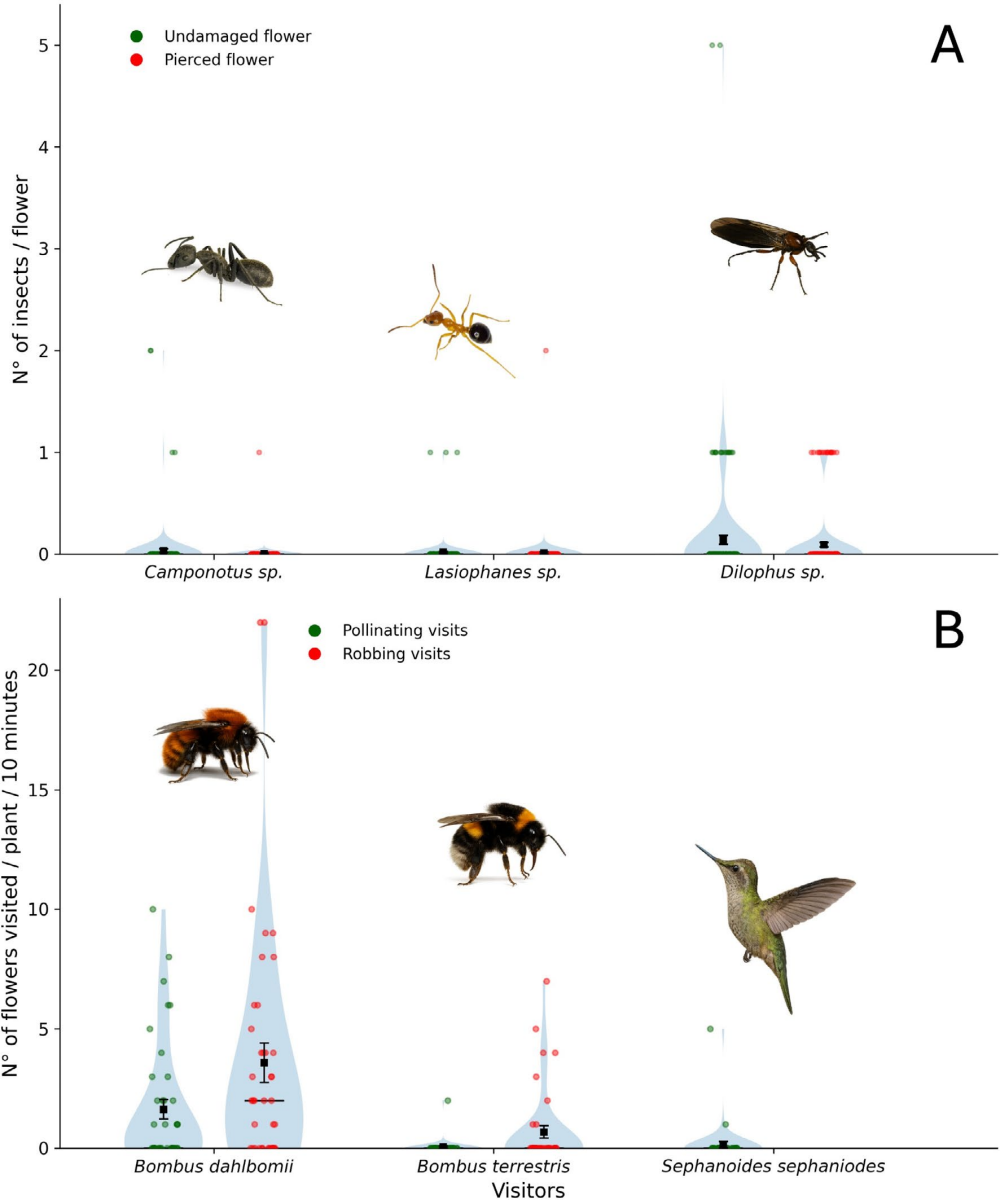
Insects recorded inside floral tubes and floral visitation patterns in *Desfontainia fulgens* . (A) Violin plots showing the number of insects per flower for three taxa recorded inside undamaged and pierced flowers. Points represent individual flowers, and squares with error bars show mean ± SE. (B) Violin plots showing the number of flowers visited per plant during 10‐min observation periods by the main floral visitors, separated by foraging strategy. Points represent plant‐level observations, and squares with error bars show mean ± SE. Green points indicate undamaged flowers in panel A and pollinating visits in panel B; red points indicate pierced flowers in panel A and robbing visits in panel B. Images illustrate the insect taxa and floral visitors recorded in each panel.

Three native insect taxa were recorded within the floral tubes of *D. fulgens* during the study: ants of the genera *Camponotus* sp. and *Lasiophane*s sp. (Formicidae), and flies of the genus *Dilophus* sp. (Bibionidae). These insects were observed on both undamaged and pierced flowers (Figure 4A). Insect abundance per flower differed significantly among taxa (Wald chi‐square = 10.13, df = 2, *p* = 0.006), whereas floral perforation status alone had no significant effect (Wald chi‐square = 2.63, df = 1, *p* = 0.105). The interaction between floral status and insect taxon was not significant (Wald chi‐square = 1.13, df = 2, *p* = 0.568). All insects recorded within flowers were small‐bodied relative to flower size and were observed moving within the corolla tube without contacting the anthers or stigma. Although pollen transfer by these insects was not directly quantified, their size and behavior suggest that they are unlikely to function as effective pollinators. Instead, they more plausibly act as nectar thieves, exploiting floral resources without contributing to pollination. This interpretation should nevertheless be considered tentative because it is based on morphological and behavioral observations rather than direct measurements of pollen deposition.

Three floral visitor species were recorded on *D. fulgens* during the observation period: the native bumblebee *B. dahlbomii* , the invasive bumblebee *B. terrestris* , and the hummingbird *S. sephaniodes* . Visitors exhibited two foraging strategies: legitimate pollination, in which nectar was accessed through the corolla opening with contact with reproductive organs, and nectar robbing, in which nectar was accessed through perforations at the base of the corolla without contact with sexual structures. *Bombus dahlbomii* acted as both a primary and secondary nectar robber, whereas *B. terrestris* acted exclusively as a secondary nectar robber. Field observations indicated that the formation of new perforations by *B. dahlbomii* often required repeated visits to the same flower. Both bumblebee species also engaged in legitimate pollination, whereas *S. sephaniodes* was observed exclusively as a legitimate pollinator.

Floral visitation rates to *D. fulgens* differed significantly among visitor species and between foraging strategies (Figure 4B). The negative binomial generalized linear model showed a strong effect of visitor species on visitation intensity (Wald chi‐square = 41.31, df = 2, *p* < 0.001) and a significant effect of foraging strategy, with nectar‐robbing visits involving more flowers per plant than legitimate pollination visits (Wald chi‐square = 8.59, df = 1, *p* = 0.003). The visitor species × foraging strategy interaction was not significant (Wald chi‐square = 4.99, df = 2, *p* = 0.082). Both bumblebee species showed higher mean numbers of flowers visited per plant during nectar robbing than during pollination, whereas *S. sephaniodes* was observed only as a legitimate pollinator. The model had a pseudo‐*R* ^2^ of 0.56, indicating a relatively good fit and suggesting that visitor identity and foraging strategy captured substantial variation in flower‐level visitation intensity.

At the plant level, the proportion of robbed flowers did not predict hummingbird visitation rate (negative binomial GLM: *β* = −0.840, *p* = 0.706). Total bee visitation was positively associated with hummingbird visitation in the count model (negative binomial GLM: *β* = 0.201, *p* = 0.004), although this result should be interpreted cautiously because hummingbird visits were recorded on only two of the 40 observed plants. When hummingbird visitation was analyzed as presence/absence, the association with total bee visitation was not statistically significant (binomial GLM: *β* = 0.136, *p* = 0.119).

### 3.4 Fruit and Seed Production

**FIGURE 5:**
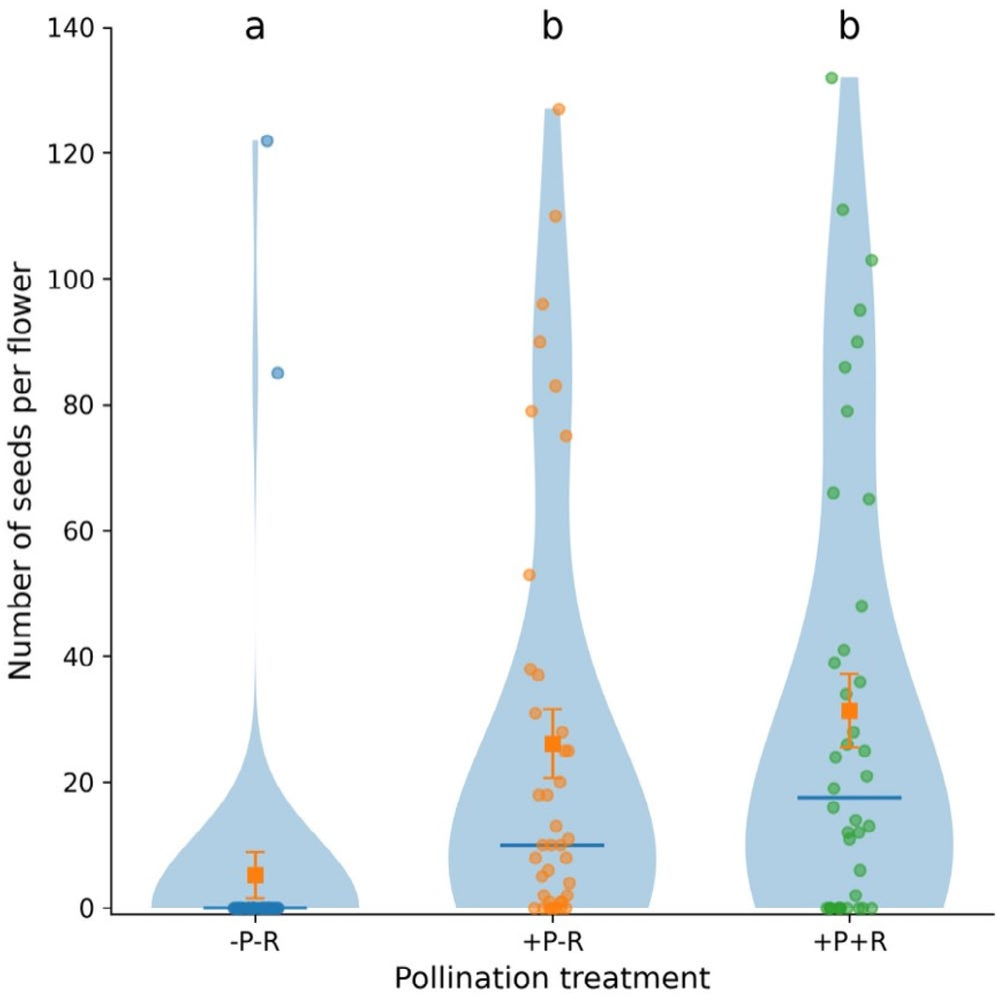
Seed production of *Desfontainia fulgens* under different pollination and nectar‐robbing treatments. Violin plots show the distribution of the number of seeds produced per flower in three experimental treatments: Pollinator exclusion without nectar robbing (−*P* − R), pollinator access without nectar robbing (+*P* − R), and pollinator access with nectar robbing (+*P* + R). Points represent individual flowers, horizontal lines inside violins indicate medians, and squares with error bars show mean ± SE. Different lowercase letters indicate significant differences among treatments.

Seed production differed among experimental treatments (Figure 5). Using a two‐part (hurdle) modeling approach, the probability of seed production was significantly higher in flowers exposed to pollinators, both in the absence (+Pol − Rob; 32 of 40 flowers) and presence (+Pol + Rob; 28 of 40 flowers) of nectar robbers, compared to flowers from which pollinators were excluded (−Pol − Rob; 2 of 40 flowers) (binomial GEE, treatment effect: Wald chi‐square = 30.56, df = 2, *p* < 0.001). No difference in seed production probability was detected between pollinator‐exposed flowers with and without nectar robbers (+Pol + Rob vs. +Pol − Rob: Wald chi‐square = 1.00, df = 1, *p* = 0.317).

Conditional on seed production, the number of mature seeds per flower also differed among treatments (negative binomial GEE, treatment effect: Wald chi‐square = 56.59, df = 2, *p* < 0.001). However, this result was strongly influenced by the very small number of −Pol − Rob flowers that produced seeds (*n* = 2), so conditional seed counts for that treatment should be interpreted cautiously. Importantly, seed number did not differ between flowers exposed to pollinators in the absence (+Pol − Rob) versus presence (+Pol + Rob) of nectar robbers (+Pol + Rob vs. +Pol − Rob: Wald chi‐square = 1.54, df = 1, *p* = 0.215).

## 4 Discussion

This study evaluates the ecological consequences of nectar robbery in *D. fulgens* , integrating flowering phenology, floral rewards, floral visitors, and reproductive output. Nectar robbery was tightly synchronized with flowering phenology and intensified during periods of high floral availability. Primary robbing strongly depleted nectar standing crops, whereas sugar concentration changed only weakly. Small ants and flies were recorded inside floral tubes, but their abundance was not significantly related to corolla perforation and they are unlikely to be effective pollinators. Bumblebees visited more flowers per plant during nectar robbing than during legitimate pollination, but this pattern describes differences among foraging modes rather than demonstrating that robbing caused a behavioral shift. Despite pronounced effects on floral rewards, nectar robbery did not reduce seed production when pollinators were present. Overall, nectar robbery in *D. fulgens* exerts strong proximate effects on floral rewards but neutral consequences for female reproductive success.

Nectar robbery in *D. fulgens* closely tracked flowering phenology, increasing in frequency during the peak flowering period. The strong temporal correlation between floral abundance and robbery frequency suggests that nectar robbers may track resource availability, rather than exploiting flowers purely opportunistically (Irwin and Maloof 2002; Almeida et al. 2022). This pattern is consistent with resource‐tracking behavior reported for nectar robbers in other hummingbird‐pollinated systems (Lara and Ornelas 2001; Irwin and Maloof 2002; Almeida et al. 2022). However, because the phenological analysis was based on iNaturalist records pooled across the species range, it should be interpreted cautiously. Population‐level variation in flowering time is likely in *D. fulgens* because populations occur across broad latitudinal and elevational gradients in Chile and Argentina. Thus, our analysis supports a coarse‐scale seasonal association between flowering and robbing, but it does not test whether every population exhibits the same flowering peak or the same intensity of nectar robbery.

Primary nectar robbing caused a dramatic reduction in nectar standing crop, with perforated flowers containing nearly an order of magnitude less nectar than undamaged flowers. This result confirms that corolla perforation by *B. dahlbomii* substantially erodes floral rewards available to legitimate pollinators, as has been documented in other ornithophilous plants (Traveset et al. 1998; Magrach et al. 2012). In contrast, nectar sugar concentration was only weakly affected and did not differ significantly between pierced and undamaged flowers. This suggests that nectar robbing primarily alters nectar quantity rather than nectar quality, likely because sugar concentration is physiologically regulated and less sensitive to short‐term nectar removal (Nicolson 2007; Pacini and Nepi 2007; Maloof and Inouye 2000; Irwin et al. 2010). Together, these findings indicate that nectar robbing reshapes the energetic landscape of flowers mainly through depletion of nectar volume, a change that is expected to influence subsequent foraging decisions by floral visitors.

Floral perforation did not significantly increase the abundance of insects within the corolla tube, although insect presence differed among taxa. Ants and flies were observed in both undamaged and pierced flowers, suggesting that flowers provide shelter or opportunistic resources independently of robbing status (Faegri and van der Pijl 1979; Kevan and Baker 1983; Willmer 2011). The absence of a perforation effect contrasts with systems where nectar robbing facilitates access by secondary visitors (Irwin et al. 2010), indicating that in *D. fulgens* corolla perforation does not substantially alter the use of flowers by non‐pollinating insects. This result suggests that indirect community‐level effects of nectar robbing via facilitation of secondary visitors are weak or context‐dependent in this system.

Nectar robbing visits in *D. fulgens* were associated with higher numbers of flowers visited per plant by bumblebees than legitimate pollination visits. This pattern is consistent with exploitative foraging strategies described for nectar robbers, where repeated visits may reflect the use of locally predictable resources rather than effective pollen transfer (Maloof and Inouye 2000; Irwin and Maloof 2002; Leadbeater and Chittka 2008; Irwin et al. 2010). However, because we did not experimentally compare the same visitors before and after robbing or quantify between‐plant movements, we do not infer that nectar robbery caused a change in visitor behavior. Instead, our data show that the two foraging modes differed in the number of flowers visited per plant.

In the case of *B. dahlbomii* , the near‐exclusive prevalence of secondary robbing indicates that elevated visitation intensity arises from efficient, learning‐based exploitation of already‐perforated flowers rather than from mechanical constraints associated with primary perforation. Experimental and observational studies have shown that bumblebees can rapidly learn and socially transmit nectar‐robbing behaviors, leading to non‐random and spatially clustered patterns of exploitation (Leadbeater and Chittka 2008). Such processes likely contribute to the high visitation rates associated with robbing observed in *D. fulgens* .

The low visitation rates of the hummingbird *S. sephaniodes* to *D. fulgens* should also be interpreted cautiously. Nectar robbery strongly reduces nectar volume in individual flowers, and studies on *F. magellanica* have shown that hummingbirds can reduce visitation to plants or flowers with depleted nectar standing crops (Valdivia, Orellana, and Murúa 2025). Nevertheless, our study did not experimentally quantify hummingbird visitation rates in the presence and absence of nectar robbery, and the population‐level frequency of robbed flowers was moderate. Therefore, we cannot conclude that nectar robbery was the main determinant of hummingbird visitation rates in *D. fulgens* . In addition, our plant‐centered 10‐min observations may underestimate hummingbird use of the population because hummingbirds commonly visit few flowers per plant before moving to another plant, whereas bumblebees often visit several flowers within a single shrub. The observed low hummingbird visitation should therefore be understood as a low per‐plant visitation rate during our standardized observation periods, not necessarily as low population‐level hummingbird activity.

The additional plant‐level analyses further support this cautious interpretation. The proportion of robbed flowers per plant did not predict hummingbird visitation, indicating that plants with a higher frequency of robbed flowers were not visited less frequently by *S. sephaniodes* during the observation periods. Total bee visitation was positively associated with hummingbird visitation in the count model, but this pattern should be interpreted cautiously because hummingbird visits were recorded on only two of the 40 observed plants and the association was not significant when hummingbird visitation was analyzed as presence/absence. These results do not support a detectable plant‐level pathway by which nectar robbery or bee activity reduced hummingbird visitation in *D. fulgens* .

Consistent with these plant‐level analyses, nectar robbery had no detectable negative effect on seed production when pollinators were present. Seed production was overwhelmingly determined by pollinator access, with flowers exposed to pollinators producing substantially more seeds than flowers from which pollinators were excluded. Importantly, seed set probability and seed number per flower did not differ between flowers exposed to pollinators with or without nectar robbers. This result aligns with recent meta‐analytic evidence showing that floral larceny commonly reduces nectar rewards but does not always translate into reduced seed production (Leal et al. 2025; Zhong et al. 2025). In *D. fulgens* , effective pollination by legitimate visitors appears sufficient to buffer the reproductive consequences of nectar robbery.

Our results reinforce the emerging view that nectar robbery should not be assumed to be universally antagonistic to plant reproduction. In *D. fulgens* , nectar robbery substantially erodes floral rewards but does not compromise female reproductive output under natural pollination conditions. These findings highlight the importance of distinguishing between proximate effects on floral traits and ultimate effects on plant fitness. In hummingbird‐pollinated systems with efficient pollinators, nectar robbery may represent a tolerable cost rather than a reproductive constraint. More broadly, our study emphasizes the need for integrative approaches that link phenology, floral rewards, visitor assemblages, plant‐level interaction patterns, and reproduction to accurately assess the ecological and evolutionary consequences of floral larceny.

## Author Contributions

**Carlos E. Valdivia:** conceptualization (lead), data curation (lead), formal analysis (lead), funding acquisition (lead), investigation (lead), methodology (lead), project administration (lead), resources (lead), software (lead), supervision (lead), validation (lead), visualization (lead), writing – original draft (lead), writing – review and editing (lead). **José I. Orellana:** data curation (supporting), investigation (equal), methodology (supporting), validation (equal), writing – original draft (supporting).

## Funding

This research was funded by the Regular Internal Research Project R07/18 from the Dirección de Investigación, Universidad de Los Lagos, and by FONDECYT project 11110230.

## Ethics Statement

This study was strictly observational and involved no capture, handling, restraint, marking, collection, or experimental manipulation of animals. All observations of birds and insects were conducted at a distance (approximately 3–4 m) and did not result in any detectable changes in animal behavior. In accordance with Chilean national regulations, non‐invasive observational studies of free‐ranging wildlife do not require institutional animal care approval or permits. All reasonable measures were taken to minimize disturbance and ensure animal welfare throughout the study. No animal was harmed or subjected to any procedure during the research (Field et al. 2019).

## Conflicts of Interest

The authors declare no conflicts of interest.

## Data Availability

The data and scripts supporting this study are available in the associated Zenodo repository (https://doi.org/10.5281/zenodo.20469376). The repository includes a plain‐text README.txt file describing the repository contents and the filters used to retrieve iNaturalist data, including filtering by plant taxon ( *Desfontainia fulgens* ) and country (Chile and Argentina).
